# Supplementary material for: Are carbon emissions trading and green financial instruments synergistic? -Comprehensive quantitative research based on content analysis
Source: PLoS One. 2024 Mar 7;19(3):e0298601. doi: 10.1371/journal.pone.0298601 (PMC10919601; doi:10.1371/journal.pone.0298601)
Supplement: S1 Data — (ZIP) [file pone.0298601.s001.zip › Data/地级市Z_F工作报告.zip!/║■▒▒/╛ú├┼╩╨/└┤╫╘╣1⁄2╓┌║┼í╛┬φ┐╦╩2╛▌═°í┐_2006.docx]

各位代表：

　　现在，我代表市人民政府向大会作政府工作报告，请连同《荆门市经济和社会发展第十一个五年规划纲要（草案）》一并审议，并请市政协各位委员和其他列席人员提出意见。

　　一、“十五”时期经济社会发展的回顾

　　“十五”时期，是荆门经济社会发展较好、改革开放步伐加快、城乡面貌变化显著、人民群众得到实惠较多的一个时期。五年来，在省委、省政府和市委的正确领导下，全市人民高举邓小平理论和“三个代表”重要思想伟大旗帜，坚持以科学发展观统领经济社会发展全局，大力实施“兴工富市”战略，解放思想，锐意改革，开拓进取，团结奋进，较好地完成了“十五”计划确定的主要预期目标，为“十一五”时期发展奠定了坚实基础。

　　（一）经济持续稳定增长。五年间，全市生产总值年均增长9.5%，人均生产总值10300元。地域财政收入由16.9亿元增加到24.6亿元，按可比口径年均增长8.4%。工业经济快速发展，规模以上工业增加值年均增长15.7%，高新技术产业增加值年均增长12.4%。全市亿元企业43家，中石化荆门分公司销售额突破百亿元，京山轻机、湖北洋丰等一批企业不断发展壮大，金龙泉啤酒两次荣获中国名牌产品称号。农业结构调整步伐加快，粮食总产稳定在200万吨左右，优质农产品基地300多万亩，畜禽养殖小区41个，“三品”标志产品88个，规模以上农业产业化龙头企业95家，日月油脂、洪森实业进入国家级龙头企业行列，累计转移农村劳动力36.8万人。县域经济发展势头强劲，县、市、区发展速度加快，效益提高，京山经验在全省推广。旅游、房地产、金融、信息、现代物流等服务业加快发展。全市社会消费品零售总额年均增长9.8%。发展后劲逐步增强，荆门热电厂三期扩建、中粮祥瑞等一批重点项目建设进展顺利。累计完成全社会固定资产投资379.5亿元，年均增长10.2%。

　　（二）改革开放取得重大进展。国有（集体）企业改革深入推进，荆襄公司改革脱困取得阶段性成果，大峪口公司顺利开工，宏图飞机制造厂破产程序终结，东光、凯龙等企业改革改制有序进行。国有公用事业单位改革逐步展开，中省企业社会职能剥离工作基本完成。积极探索适应新时期发展要求的国资监管体制。多种所有制经济共同发展，“十五”末非公有制经济的比重达39%。商品市场和资本、信息、技术、人才等要素市场较快发展。整顿规范市场经济秩序，市场物价监管、食品药品监管、质量技术监督和烟草专卖等工作得到加强。农村税费改革成效显著，已取消农业特产税、免征农业税。乡镇综合配套改革稳步推进，乡镇党政机构、事业单位改革和国有农场综合改革基本完成。顺利完成市、县两级政府机构改革。深化行政审批制度改革，取消和下放审批项目近1000项。积极推进财政管理体制改革，基本建立起比较规范的市直部门预算、国库集中收付、政府采购和收支两条线管理制度。信用环境建设不断深化，我市连续三年被评为湖北省“A级金融信用市州”。投资、流通和国土资源管理体制等改革取得新进展。对内对外开放不断扩大，累计吸收外商投资2.8亿美元，年均增长44.4%；完成出口总额3.5亿美元，年均增长11.1%。

　　（三）基础设施建设和城市建设迈出新步伐。累计完成基础设施建设投资150亿元，比“九五”时期增长2倍以上。全市新增公路通车里程2309公里。襄荆高速公路、长荆铁路、江汉航线投入营运，荆宜、随岳、武荆高速公路荆门段开工建设，二级公路路网建设基本完成。电力“两网”改造结束。固定电话和移动电话用户分别达到48.7万户和78万户，比“九五”末增长1倍和17.2倍；互联网用户9.8万户，增长3.9倍。汉江堤防建设不断加强，除险加固259座水库。城镇化进程加快，城市化水平为38.6%。南城区框架初步形成，中心城区建成区面积达到44.6平方公里，人口38.1万人。建成开放天鹅广场、月亮湖广场、东宝广场，打通东宝山隧道，新建和改造象山大道、长宁大道、白云大道等55条道路，建设天然气管网、三水厂管网、夏家湾污水处理厂等公用设施项目，城区生活污水集中处理率、垃圾无害化处理率分别达到51%和75%。钟祥、京山、沙洋县城和一批重点小城镇建设成效明显。环境保护和生态建设取得积极进展，全市森林覆盖率达到35.7%，比“九五”末提高2.5个百分点。加大综合整治力度，努力建立城市长效管理机制。漳河水源保护工作不断加强，青山水泥厂已经关闭，竹皮河城区段经过治理成为一道亮丽的景观。

　　（四）社会事业全面进步。坚持实施科教兴市战略，科技创新体系逐步建立，荆门经济开发区（荆门高新区）建设取得实质性进展，全市高新技术企业24家，我市连续三次被授予全国科技进步先进城市称号。推进教育结构调整和教育体制改革，基础教育、职业教育、成人教育协调发展。五年间，筹集资金6917万元，改造农村中小学危房19.5万平方米；筹集资金3100万元，资助12万余名贫困学生。本科学院筹建工作进展顺利。全民健身活动广泛开展，竞技体育取得突破，何军权在雅典残奥会上勇夺4枚金牌。文化事业和文化产业加快发展，大型现代花鼓戏《闹龙舟》获中宣部“五个一”工程奖，《十二月等郎》入选全国舞台艺术精品工程30台初选剧目。成功举办第五届中国民间艺术节等重大文化活动。广播电视实现行政村“村村通”。建成青少年活动中心、妇女儿童活动中心和青少年科技馆。市、县、乡三级疾病预防控制、医疗救治和卫生监督执法体系建设取得新进展，新型农村合作医疗试点工作已经启动。成功应对“非典”疫情，有效防控高致病性禽流感，艾滋病、结核病、血吸虫病等重大传染病和地方病的防治得到加强。人口保持低生育水平，人口自然增长率由“九五”末的3.2‰下降至1.8‰。民族、宗教、对台、外事、侨务、人防、驻外联络、气象、档案、方志、保密、老龄、残疾人等各项工作取得新成绩。

　　（五）人民生活继续改善。城镇居民人均可支配收入达到8585元，年均增长8.5%。农民人均纯收入由2980元增加到3738元。居民人均储蓄存款6855元，比“九五”末增长1.1倍。城镇居民用于教育、文化、旅游等方面的支出明显增加，人均居住面积由12.6平方米增加到25.1平方米。累计有7.1万人次下岗失业人员实现再就业，城镇登记失业率控制在4.2%以内。基本养老、失业、医疗、工伤和生育保险参保人数分别为18.2万、15.3万、24.2万、13.1万和11.9万。城市低保、特困群众救助、城乡贫困群众医疗救助和住房公积金制度不断完善。柴湖、漳河库区扶贫取得明显成效。农村低收入贫困人口减少15.6万人，“五保户”集中供养率达88%。农村“改水”受益面达84.1%。2004年、2005年市政府承诺的改善城乡人民生活的实事顺利完成。

　　（六）精神文明建设和民主法制建设不断加强。坚持用邓小平理论、“三个代表”重要思想武装广大干部群众，认真开展保持共产党员先进性教育活动。深入贯彻《公民道德建设实施纲要》，扎实开展文明城市、文明村镇、文明单位创建活动，公民素质和城乡文明程度进一步提高。我市荣获中国优秀旅游城市和省级文明城市、园林城市、卫生城市称号。加强基层民主政治建设，健全村务公开和村民自治制度，完善城市社区居民自治和基层管理体制。顺利完成第六届村委会换届选举工作。深入开展普法教育。坚持依法行政，推进依法治市，认真接受法律监督、民主监督和舆论监督，定期听取人大代表、政协委员的意见和建议，各项人大议案、代表建议和政协提案全部办复。全面推行政务公开，成立行政服务、国库集中收付、招投标交易、电子政务、经济发展环境投诉等“五个中心”，开通“12345”市长专线、“中国.荆门”政府门户网站，建立政府信息公开和新闻发布制度。加强和改进信访工作，积极化解各类矛盾。高度重视安全生产，公共安全管理得到加强。强化社会治安综合治理，依法严厉打击各种犯罪，全力推进“平安荆门”建设。积极支持军队建设，大力开展国防教育和“双拥”活动，国防后备力量建设得到加强，军政、军民关系更加融洽。加大源头治腐力度，强化行政监察、审计监督，廉政建设进一步加强。

　　各位代表，“十五”时期我市改革开放和现代化建设取得的成绩，是全市人民共同奋斗的结果，是市委正确领导、历届政府打下良好基础和人大、政协监督支持的结果，是中国人民解放军驻荆部队、武警官兵和各民主党派、工商联、无党派人士、人民团体大力支持的结果。在此，我代表市人民政府，向全市各条战线的广大干部群众，向所有关心和支持我市经济社会发展的社会各界人士，表示崇高的敬意和衷心的感谢！

　　在充分肯定成绩的同时，我们也清醒地看到，当前我市经济社会生活中还存在不少问题和困难，主要是：结构性、体制性、机制性矛盾依然突出，改革调整的任务艰巨；经济发展不够协调平衡，运行质量和效益不高，增长方式比较粗放，资源约束和环境压力加大；农业基础脆弱，农民增收的长效机制尚未形成；财源结构不尽合理，财政状况与经济发展的速度和社会发展的需求不相适应；社会事业发展相对滞后，就业再就业任务繁重，社会保障体系还不够健全，就医、入学中的一些问题未能得到较好解决，部分群众生活比较困难，社会不稳定因素依然存在；政府自身改革和职能转变尚未到位，社会管理和公共服务职能较弱，管理方式、办事效率、服务质量还不能较好地适应新形势的要求，还存在着官僚主义、形式主义以及腐败现象，等等。对这些问题，我们一定要高度重视，采取切实有效措施，认真加以解决。

　　二、“十一五”时期的发展目标和主要任务

　　今后五年是荆门至关重要的发展时期。综合考虑发展阶段、发展环境和基础条件，市政府组织编制了《荆门市经济和社会发展第十一个五年规划纲要（草案）》，提出了“十一五”时期全市经济社会发展的指导思想和发展目标。指导思想是：以邓小平理论和“三个代表”重要思想为指导，以科学发展观统领经济社会发展全局，抢抓国家促进中部地区崛起的政策机遇，坚持以加快发展为主题，以调整经济结构、转变增长方式、提高增长质量为主线，以改革开放和自主创新为动力，以改善城乡人民生活为根本出发点，着力推进“兴工富市”战略实施，着力推进工业化、城镇化、农业现代化，着力推进社会主义新农村建设，促进经济社会全面、协调、可持续发展，促进城乡居民收入稳定增长，加快构建和谐荆门。通过五年的努力，使全市综合经济实力明显增强、工业化水平明显提升、城镇化进程明显加快、人民生活水平明显提高、生态环境明显改善、社会发展明显进步。主要发展目标是：生产总值年均增长10%以上，一、二、三产业分别增长3.5%、13.5%和11%；地域财政收入年均增长11.5%；全社会固定资产投资年均增长11%；外贸出口年均增长20%，累计吸收外商投资6亿美元以上；单位生产总值综合能耗比“十五”末下降20%；城市化水平达到45.5%，中心城区建成区面积达54平方公里、人口达50万人、人均公共绿地面积达11.7平方米，全市森林覆盖率达到38%以上；城镇居民人均可支配收入年均增长8%，农民人均纯收入年均增长6%。

　　确定上述指导思想和主要目标，充分体现了科学发展和建设和谐荆门的要求，充分体现了省委、省政府对荆门发展的要求，充分体现了市委关于经济社会发展的总体思路和全市人民的共同愿望，充分体现了加快发展的客观需要和现实可能。《纲要（草案）》贯穿了“四个统一”的原则要求，即坚持把科学发展与加快发展统一起来，把走新型工业化道路与建设社会主义新农村统一起来，把改革创新与稳健务实统一起来，把经济发展与社会发展、人的全面发展统一起来，抢抓机遇，乘势而上，推动荆门经济社会又快又好地发展。《纲要（草案）》及《说明》已印发给各位代表，请大家审议。这里，着重报告“十一五”时期全市经济社会发展的六个重点：

　　（一）加快经济结构战略性调整。

　　一是全面增强工业核心竞争力。坚持走新型工业化道路，壮大优势行业，提升传统行业，培植新兴行业，努力把荆门建设成为湖北的化学工业基地和建材工业基地。石化工业：充分发挥石化工业的支撑作用，支持石化企业稳步扩大原油加工能力，积极发展石化产品深加工，延伸产业链条，带动地方工业发展。磷化工业：合理开发和利用磷矿石资源，实行采选、矿肥、矿化结合，做强做大骨干企业，推进磷化工向精细化工方向发展。建材工业：提高新型干法水泥比重，发展新型建材产品。机械工业：推进机电一体化技术应用，发展特色机械产品。纺织工业：加快棉纺新技术应用和纺织全流程更新改造，发展纺织制品和服装加工业。食品工业：围绕粮、油、肉、鱼、菜、果等农产品深加工，推动食品加工由初加工向精深加工转变。高新技术产业：重点支持生物医药、新材料工业和电子信息业发展。加快建设荆门经济开发区（荆门高新区），促进钟祥、京山、沙洋经济开发区和东宝工业集中区发展，办好磷化工业园。实施“四个一”工程，支持1家企业（中石化荆门分公司）销售收入过200亿元，1家企业（湖北洋丰集团）过100亿元，1家企业（京山轻机集团）过50亿元，葛洲坝水泥厂、百科药业、金龙泉集团等10家企业过10亿元。同时，大力扶持和加快中小企业发展，培育一批销售收入过5亿元的骨干民营企业，培育3个以上“中国名牌”产品或“中国驰名商标”。

　　二是奋力推进社会主义新农村建设。按照“生产发展、生活宽裕、乡风文明、村容整洁、管理民主”的要求，坚持把解决好“三农”问题作为工作的重中之重，从实际出发，科学规划，分步实施，以发展农村生产力、促进农民增收为重点，加快农村交通、水利、高产农田等基础设施建设，着力解决生产生活中最迫切的问题，充分调动广大农民群众建设新农村的积极性。大力发展现代农业，加快农业标准化、规模化生产，稳定粮食生产，开发高效棉田，适当扩大“双低”油菜面积，发展优质蔬菜和林果业基地。加快发展畜牧业和水产业，力争养殖业占农业增加值的比重达到60%。深入推进农业产业化经营，培植优势产业带和龙头企业，突破性发展农产品加工业，扶持国宝桥米、中粮祥瑞、陈湾猪场等龙头企业发展壮大，支持国宝桥米创“中国名牌”，力争到2010年全市粮食、肉类等农产品加工能力比2005年翻一番，有20家以上种、养、加龙头企业的销售收入过亿元、5家过5亿元。推进科技兴农，全面实施“种子工程”和“沃土工程”，加快农机化发展。加强农村路网、电网、广播电视网、信息网和水利基础设施建设，实施“一建三改”（建沼气池，改厨、改厕、改圈）工程，沼气综合利用户达60%以上。以列入全省“百镇千村”的4个镇、59个村为重点，积极开展村容村貌综合整治，改善农村生活环境。大力发展农村教育、文化、卫生、体育等公共事业，加大农民教育培训力度，倡导健康文明新风尚，培育造就新型农民。继续推进农村九年义务教育改革和县乡财政体制改革等农村各项改革。深化国有农场改革。严禁新增并稳妥化解乡村债务。实施“阳光工程”，引导农村富余劳动力向非农产业和小城镇转移，五年新增转移农村劳动力10万人以上。着力抓好扶贫开发工作，促进贫困农民尽快脱贫。加强农村基层民主政治建设，确保广大农民群众依法行使当家作主的权利。

　　三是加快发展和提升服务业。改造、提高传统服务业，推行连锁经营、物流配送、代理制等现代流通方式，着力培育一批商贸、零售、餐饮骨干企业。大力发展现代服务业，加强物流园区和物流基地建设，完善现代物流服务体系；建立多种形式的融资平台和信用担保体系，促进金融保险业发展；推进宽带通信网、数字电视网和下一代互联网的建设与融合，发展电子政务、电子商务，加快“数字荆门”建设。积极培育成长型服务业，以明显陵.大洪山、城区旅游、纪山楚文化为重点，加快旅游业发展，力争全市旅游经济总收入年均增长16%以上；引导房地产业健康发展，完善住房保障制度，建立适应荆门实际的经济适用房、廉租住房供应体系，提高城镇居民居住质量；健全住房公积金制度，切实缓解中低收入家庭的住房困难；扶持社区服务业、文化产业、中介服务业做大做强。

　　（二）着力推进改革开放。一是深化行政管理体制改革。加快转变政府职能，继续推进政企分开、政资分开、政事分开、政府与中介组织分开。完成事业单位体制改革。二是深化国有企业和国有资产管理体制改革。加大国有独资企业和垄断行业的改革力度，促进投资主体多元化，加快建立规范的现代企业制度。推进国有企业改革向纵深发展，妥善解决改制企业遗留问题。深化市政公用事业改革，开放市政公用事业资本市场、经营市场和作业市场。进一步完善国有资产监管体制。按照平等待遇、开放领域、引导产业、调整结构、创新服务的思路，鼓励、支持和引导非公有制经济发展。三是深化财政和投资体制改革。加快公共财政体系建设，继续深化部门预算、国库集中收付、政府采购和收支两条线等财政管理制度改革。规范政府投资管理行为，建立责任追究制，推行政府投资项目代建制，健全非政府投资项目核准与备案制。四是提高利用外资的质量和水平。进一步改善投资环境，健全投资服务体系，积极运用股权融资、项目融资、并购、境外上市等方式，努力扩大利用外资规模。稳步推进商贸、金融、科技、文化、教育、旅游等第三产业的对外开放。五是增强出口竞争力。转变外贸增长方式，优化出口商品结构，加强机电产品、农副产品、矿产品、轻工产品和纺织服装等出口基地、出口品牌建设，培育一批年出口额过千万美元的龙头出口企业。六是加强区域经济技术合作。开展“东引西进”，积极拓展与武汉等大城市的合作与交流。

　　（三）促进城镇化和区域经济健康发展。努力建设鄂中区域中心城市、新兴特色工业城市、历史文化名城和生态宜居城市。一是加快中心城区建设。严格执行荆门城市总体规划，重点建设南城区，适度改造北城区，不断增强中心城区辐射带动力。完善北城区西片商业文化和综合服务功能，突出北城区东片以石油化工和电力工业为主的传统工业地位。坚持把南城区建设与城区工业发展紧密结合，力争“十一五”末将南城区建设成为市级行政中心和以高新技术产业为主的新城区，打造现代化城市格局。加强城市管理，健全长效管理机制。充分挖掘历史文化资源，提升城市文化品位。二是大力发展县域经济。完善钟祥、京山、沙洋城区综合服务功能，努力把京山建设成为全省重要的机械制造基地和现代农业示范基地，把钟祥建设成为全省磷化、食品、纺织工业基地和全国知名目标旅游城市，把沙洋建设成为全省重要的农产品加工和纺织工业基地，把东宝建设成为全市统筹城乡发展示范区。三是加快小城镇建设。按照构建“一个中心、两条经济带”的布局，加快发展胡集、后港、漳河、宋河等一批重点小城镇，引导发展张集、李市、曹武、马河等口子镇和绿林、客店、纪山、屈家岭等旅游镇，积极发展其他小城镇。四是加强基础设施建设。加快高速公路、城市交通、乡村公路建设，做好荆宜、武荆、随岳高速公路荆门段建设的服务工作，抓好二级公路和农村公路改建，实现村村通油（水泥）路。完善全市电力大环网，积极服务荆门热电厂扩建、碾盘山水利枢纽工程和钟祥北山抽水蓄能电站建设。推进天然气工业利用，改善能源结构。增加城区集中供暖面积。加强水利基础设施建设，抓好防洪、灌溉、饮水安全和水保生态工程建设。

　　（四）大力实施科教兴市和人才强市战略。一是全面推动科技进步。认真贯彻落实全国科技大会精神，坚持联合创新和自主创新相结合，重视企业技术中心和技术队伍建设，鼓励重点企业与大专院校共建研发机构和博士后工作站，推进产学研合作，提高自主创新能力。加强科技中介服务机构和技术创新平台建设，健全农村科技推广服务体系。多渠道加大科技投入，建立科技风险投资基金，落实科技要素入股和参与分配等政策，鼓励和引导企业增加科技创新投入。二是加强教育体系建设。全面普及和巩固九年义务教育，深入推进素质教育和创新教育，加快发展中等职业教育，促进终身教育，全民人均受教育年限达到9年。努力建成一所本科学院。大力发展农村教育，深化农村义务教育经费保障机制改革，促进城乡教育协调发展。鼓励社会力量办学，推进办学体制改革和投资体制改革，努力形成以政府办学为主体、公办和民办学校共同发展的新格局。三是加快人才资源开发。继续实施“113”人才发展计划，全面启动农村实用人才工程，加强企业家队伍建设，加强高技能人才培养，力争到2010年全市人才总量达到18.5万人。加大科技和人才开发投入力度，加强人才市场体系建设，建立健全优秀人才激励机制，争取每年吸纳各类高级人才和大中专毕业生6000人以上。

　　（五）建设资源节约型、环境友好型城市。一是大力发展循环经济和节约利用资源。重点抓好工业、建筑、交通运输和农业机械节能，积极推进机关事业单位、居民和公用设施节能。实行最严格的耕地保护制度，切实保护好基本农田，合理开发和集约利用土地资源。推广应用节水新技术、新设备。加强废弃资源综合利用，努力形成5--10个产业循环式组合链条。推行清洁生产，加强生态工业园区建设。大力发展循环农业，促进农业可持续发展。二是加大生态环境建设与保护力度。积极实施天然林保护、封山育林、荒山造林、水土保持、坡耕地治理、长湖湿地保护、生态农业示范等工程，建设“生态荆门”。加强山林管理，禁止乱砍滥伐。加强漳河等饮用水源的水体保护和渠系保护。认真抓好汉江荆门段水污染防治和竹皮河、杨树港、钟祥护城河、西荆河、京山河等水环境治理工程。严格控制工业污染，提高工业废气处理率和废水排放达标率。加强城区大气环境治理，改善空气质量。

　　（六）积极构建和谐荆门。一是千方百计促进就业。大力发展就业容量大的产业和各类所有制中小企业，健全就业服务体系，建立稳定的免费就业服务、再就业培训资金投入机制，努力解决困难群众、特殊群体的就业问题。实施就业援助制度，创建充分就业社区，促进“零就业”家庭就业。二是完善社会保障体系。进一步扩大社会保障覆盖面，逐步将非公有制企业、有条件参保的灵活就业人员、被征地农民纳入社会保障范围。增加财政对社会保障的投入，多渠道筹措社会保障基金，逐步做实个人账户。稳步提高最低生活保障和最低工资标准。完善城市低保、农村“五保户”供养和特困群众救助制度。积极发展慈善事业，促进残疾人康复和就业。贯彻男女平等基本国策，落实妇女儿童发展规划，维护妇女儿童合法权益。加强社会福利机构建设，提高社会福利水平。三是加快发展文化、体育、卫生等各项社会事业。进一步繁荣文化事业，重视发展哲学社会科学，大力发展新闻出版、文化艺术、广播电视等事业。建好市体育文化中心，扩建市博物馆、图书馆和群艺馆。加强乡镇、社区综合文化站和村级文化活动室的建设，活跃群众文化生活。积极加强文物保护工作。推进广播电视数字化。广泛开展全民健身运动，加强社区、乡镇健身设施建设。发展竞技体育，重点建好划船、举重、射击、网球等训练基地。健全公共卫生和医疗服务体系，推行新型农村合作医疗，办好惠民医院，构建社区卫生服务网络，完善城乡特困群体医疗救助体系，努力解决人民群众看病难、看病贵的问题。坚持预防为主，继续做好艾滋病、血吸虫病、结核病等重大传染病和地方病的防治工作。认真落实农村计划生育奖励扶助制度，加强计划生育特别是流动人口计划生育的管理和服务，稳定低生育水平。坚持实施出生人口性别比综合治理。四是加强精神文明建设。抓好市民文明素质教育和未成年人思想道德建设，关心下一代健康成长，预防青少年犯罪。大力弘扬以爱国主义为核心的民族精神、以改革创新为核心的时代精神，广泛开展“文明荆门”建设活动，在全社会倡导爱国守法、明礼诚信、团结友善、勤俭自强、敬业奉献的基本道德规范。五是加强民主法制建设。进一步扩大基层民主，完善村民自治、社区自治制度，积极推进乡镇“两推一选”和村委会、社区直选，继续抓好政务公开、村务公开、厂务公开、事务公开。全面推进依法治市，深入开展“五五”普法教育。促进司法体制改革，维护司法公正。积极开展法律援助，支持司法救助。认真落实信访条例，畅通群众诉求渠道，通过调解、仲裁、行政复议等多种方式，依法妥善处理各种社会矛盾。加强社会治安综合治理，维护社会稳定。建立健全社会突发公共事件应急管理体系。加大安全生产监管力度，坚决防止和减少各类重大事故，保障人民生命财产安全。重视各种自然灾害预测预报，提高防灾减灾能力。加强社区建设和管理，创建平安社区和安全文明小区。继续整顿市场经济秩序，严厉打击制假售假、商业欺诈等违法行为。强化对食品、药品等重要领域的监管，加强价格管理与监测。六是积极支持军队和国防建设。认真做好新时期“双拥”工作，争创“双拥”模范城。深入开展国防教育，加强国防动员、民兵预备役工作和人民防空建设。同时，进一步做好民族、宗教、对台、外事、侨务、档案、保密、方志等工作。

　　三、2006年的主要工作

　　各位代表，荆门经济社会发展正进入新的机遇期，“十一五”将是我市经济结构进一步调整、对外开放进一步扩大、基础设施进一步完善、发展环境进一步优化、综合实力进一步增强的关键时期。五年看三年，三年看头年。在起步之年，我们没有任何理由懈怠，必须牢固树立强烈的机遇意识、发展意识、创新意识、大局意识，切实增强加快发展的紧迫感和责任感，进一步振奋精神，坚定信心，始终保持昂扬向上的锐气、克难奋进的勇气、抢前争先的志气，立足科学发展，加快开放发展，着力创新发展，促进和谐发展，努力实现“十一五”的良好开局。今年全市经济社会发展的主要预期目标是：生产总值增长11%；规模以上工业增长18%；全社会固定资产投资增长18%；社会消费品零售总额增长13%；外商投资增长18%，外贸出口增长15%；万元生产总值综合能耗下降4.4%；地域财政收入、一般预算收入分别增长12%和11%；城镇居民人均可支配收入增长6%，农民人均纯收入增长5%；城镇新增就业2.8万人，城镇登记失业率控制在4.3%以内；人口自然增长率控制在3‰以内。实现上述目标，关键是要按照市委五届八次全会和全市经济工作会议精神，突出重点，狠抓落实。

　　（一）围绕企业增效，加快工业经济发展。大力发展城区工业，积极服务中省企业，充分发挥其带动作用。强化规划引导和政策扶持，加快荆门经济开发区（荆门高新区）建设，大力支持东宝工业集中区发展，积极筹建磷化工业园，进一步提高钟祥、京山、沙洋经济开发区的发展水平。加大项目建设力度，努力服务荆门热电厂三期扩建等11个续建项目建设，认真组织实施中粮祥瑞粮油加工扩建、凯龙集团硝酸铵等9个重点项目开工建设。抓住国内外产业梯度转移的机遇，坚持不懈地加强招商引资，创新招商方式，加大项目调研、包装、推介力度，着力引进大项目、大企业，提高项目履约率、资金到位率和投产达产率。扶持京山轻机、百科药业、普安阀门等企业扩大出口规模，实现外商投资和外贸出口“双过亿”。加快海关荆门办事处建设。加强自主创新能力建设，鼓励金龙泉、百科药业等企业创建国家级技术中心，支持新美香、格林美、日月油脂等企业创建省级技术中心，力争年内科技开发新产品达到30项以上。大力发展循环经济，推进火电、化工、建材等行业的节能节水节材，扶持30个资源综合利用项目建设。严禁上污染严重的项目，严格控制新上木材加工项目。继续加强和改善对经济运行的调节，做好煤电油运等要素资源的协调与衔接工作。

　　（二）围绕农民增收，扎实推进社会主义新农村建设。按照建设新农村和统筹城乡发展的要求，注重研究加强“三农”工作和发展现代农业的重点问题，全面落实党在农村的各项政策，努力实现农业增效、农民增收、农村发展目标。优化农业内部结构，大力发展畜牧业，加快养殖小区建设，提高水产养殖效益。强力推进农业产业化经营，立足优势资源和特色产业，培植龙头企业和精品名牌，为县域经济发展注入活力。重点扶持国宝桥米、中粮祥瑞、日月油脂、宝源木业、洪森实业等龙头企业发展壮大。调整优化农业品种结构，培育推广名特优新品种，力争农产品综合优质率提高到60%以上。完成大型商品粮生产基地建设项目前期工作。新增城郊蔬菜基地面积2000亩。进一步畅通“绿色通道”，积极发展连锁经营，推进农产品批发市场升级改造，大力发展农村专业合作经济组织，培育新型农村流通市场。突破性发展农村劳务经济，力争农民工资性收入占农民纯收入的比重比上年提高5个百分点。加强水利基础设施建设，抓好汉江堤防、各类水库除险加固、大型灌区续建配套建设，加强农村通村公路建设和管理。做好高致病性禽流感等重大动物疫病防控工作。严禁水库投肥养鱼。启动“十村示范、百村整治”的新农村建设试点，免费提供村庄住宅建设的设计方案。继续实行结对帮扶、对口帮扶和搬迁扶贫。进一步加大农业投入力度，今年市财政支农切块资金增长25%。加强农村基层文化建设，加大农民教育引导力度，严厉打击地下“六合彩”等赌博行为。

　　（三）围绕培植财源，进一步做好财税金融工作。切实把提高经济效益、促进区域协调发展与加强财源建设结合起来，不断增强综合经济实力。调整优化税源结构，重视高利税行业、企业的发展和项目建设，巩固基础财源，壮大骨干财源，培植后续财源。坚持依法治税，强化税收征管，确保财政收入稳定增长，不断提高财政收入质量。按照公共财政要求，优化支出结构，确保重点支出和调资增资政策落实，增加对社会事业、社会保障和社会公共产品的投入。做好金融服务经济社会发展工作，加大对城区工业、中小企业、县域经济发展的信贷支持，积极为各类所有制的金融机构落户荆门创造条件。推进信用企业、信用乡镇、信用社区建设，营造良好金融环境。进一步落实促进非公有制经济发展的政策，鼓励自主创业、艰苦创业、全民创业，激活民间投资，大力培育市场主体。建立和完善中小企业贷款担保体系和风险补偿机制，努力解决中小企业贷款难问题。

　　（四）围绕提升城市功能，加快城市建设与发展。坚持高起点规划、高品位建设、高标准配套，全面推进南城区建设，认真谋划、起步实施一批重点建设项目。着力完善城市综合服务功能，继续抓好北城区环境改造、市政设施配套和绿化上档升级。大力发展现代服务业，抓好城区旅游、明显陵.大洪山、京山温泉、纪山楚文化等旅游项目建设，支持商贸企业做强做大，促进物流业加快发展。加强普通商品房、经济适用房建设与管理，培育住房、汽车、文化、教育、体育等消费热点。做好本科学院筹建工作。确保市体育文化中心主场馆按时保质竣工。加大城市管理力度，综合整治城市环境，继续抓好漳河水源保护工作，切实加强粉尘、噪音等污染治理，提高城区空气质量，改善城市人居环境。加快县城和重点镇建设，引导农村人口向城镇集中、工业企业向园区集中、生活居住向小区集中。

　　（五）围绕改善群众生活，努力为民办实事。坚持以人为本，按照“打基础、管长远”和财力向基层倾斜的要求，加大投入力度，着力解决人民群众最关心、最直接、最现实的利益问题，不断提高城乡居民收入水平和生活质量。今年，市政府将重点办好十件实事：（1）筹集560万元资金，解决1400户、4800人城镇无生活来源的“零就业家庭”就业和生活保障问题。（2）筹集1000万元资金，资助义务教育阶段3万名贫困学生享受“两免一补”，资助100名大学贫困新生入学，资助139名残疾学生免费就读市特殊学校，资助1500名贫困家庭初中毕业生上中职学校。（3）筹集850万元资金，救助农村特困人口4.2万人，将农村孤儿全部纳入救助范围，继续对城镇“低保”对象、“五保户”、农村特困户、特困优抚对象中患重大疾病的人员实施医疗救助，资助3.5万名特困户、“五保户”参加新型合作医疗，新增参合农民80万人以上。（4）加强农村生态家园建设，筹集400万元资金，扶持5000个农户实施“一建三改”工程。（5）筹集2000万元资金，继续推进重点贫困乡（镇）、村和革命老区的扶贫开发工作。（6）完成农村劳动力培训10万人；新增转移农村劳动力4万人。（7）筹集1500万元资金，改造中心城区20条小街小巷，增加城市绿地面积30公顷。（8）筹集600万元资金，解决农村1.55万人饮水安全问题。（9）筹集5000万元资金，新建农村通村公路500公里。（10）加强乡村商业、文化设施建设，筹集400万元资金，在京山、东宝两个试点县区新建或改建乡村连锁店200家；筹集150万元资金，扶持发展农村文化中心户、村文化活动室。

　　（六）围绕行政提速、改进服务，切实加强政府自身建设。坚持科学行政、民主行政、依法行政，塑造“为民、务实、清廉”的政府形象。主动适应新形势、新任务、新要求，勤于学习，善于思考，勇于实践，努力提高贯彻科学发展观的能力、驾驭全局的能力、处理利益关系的能力和务实创新的能力。全面推进依法行政，自觉接受人大及其常委会监督，重视并支持人民政协履行政治协商、民主监督、参政议政职能，主动听取各民主党派、工商联、无党派人士的意见和建议，积极支持工会、共青团、妇联等群团组织开展工作。完善行政执法管理体制，强化行政执法责任制和责任追究制，加强行政执法监督，促进公正执法、文明执法、高效执法。加快服务型政府建设，继续深化行政审批制度改革，完善行政管理流程，规范行政许可行为。大力推行“四个公开”，积极发展电子政务，促进行政权力公开透明运行，开展网上审批试点。努力发挥市行政服务管理委员会作用，完善“五个中心”的运行机制和服务功能，切实提高办事效率和服务水平。畅通社情民意反映渠道，进一步办好“12345”市长专线和“中国.荆门”政府门户网站，健全专家咨询论证、公示听证等制度，提高科学、民主决策水平。坚持从实际出发，将中央、省委省政府加快发展的要求与荆门实际相结合，聚精会神搞建设，一心一意谋发展，把各方面积极性、创造性凝聚到加快荆门发展上来。加强政风建设和公务员队伍建设，坚持权为民所用、情为民所系、利为民所谋，求真务实，真抓实干，全心全意为人民服务。切实转变作风，反对官僚主义、形式主义，力戒空谈浮夸、做表面文章，主动为基层服务、为企业排忧、为群众解难。进一步强化政务督查、效能监察、审计监督、责任追究，确保任务分解到位、责任传递到位、工作落实到位。妥善处理各种社会矛盾，维护社会稳定，营造良好的发展环境。加强物价监管和食品药品安全监管，严格落实安全生产责任制。坚持厉行节约，反对铺张浪费，以节约型政府建设带动节约型社会建设。加强廉政建设和反腐败斗争，坚决纠正损害群众利益的不正之风，坚决查处行政“乱作为、不作为、慢作为”行为，坚决惩治腐败分子。

　　各位代表，加快荆门发展，是历史赋予我们的庄严使命，是全市人民的殷切期盼。让我们紧密团结在以胡锦涛同志为总书记的党中央周围，在省委、省政府和中共荆门市委的领导下，紧紧依靠全市人民，全面落实科学发展观，抢抓机遇，开拓创新，求真务实，团结拼搏，为圆满完成“十一五”规划的各项任务、共同创造荆门更加美好的未来而努力奋斗！
